# Supplementary material for: Potential Mechanisms of Metformin-Induced Apoptosis in HeLa Cells
Source: Biomolecules. 2023 Jun 6;13(6):950. doi: 10.3390/biom13060950 (PMC10295975; doi:10.3390/biom13060950)
Supplement: Supplementary file 1 [file biomolecules-13-00950-s001.zip › biomolecules-2409610-supplementary.pdf]

Supplementary Materials

Table S1. The list of RT-qPCR primers

Genes	Primers (5’-3’)	Size (bp)
ACTB	R: CTGGAACGGTGAAGGTGACA<br>F: AAGGGACTTCCTGTAACAACGCA	140
HRK	R: AGCGAGCAACAGGTTGGTGA<br>F: AGGCGCTGTCTTTACTCTCC	132
DDIT3	R: CCTCCTGAAAATGAAGAGGAAG<br>F: CTCTGGGAGGTGCTTGTGAC	116
CASP3	R: TTTTTCAGAGGGGATCGTTG<br>F: CGGCCTCCACTGGTATTTTA	151
BCL-2	R: GATAACGGAGGCTGGGATGC<br>F: TCACTTGTGGCCCAGATAGG	150

Table S2. Metformin for semi-inhibitory concentrations (IC_{50}) and cell viability of Hela cells

Groups/（mM） Time/h	IC_{50}	0	3	6	12	24	48
		Cell viability （Mean ± SD %）					
12	47.34 ± 9.94	100.00 ± 3.70	97.25 ± 6.53	97.14 ± 3.28	86.81 ± 13.40	76.59 ± 6.19	64.92 ± 9.07
24	35.87 ± 2.11	100.00 ± 6.36	90.26 ± 6.37	82.62 ± 10.96	76.70 ± 7.38	58.88 ± 6.43	40.58 ± 3.71
36	35.13 ± 4.20	100.00 ± 8.98	93.48 ± 7.24	95.20 ± 10.98	85.46 ± 7.61	56.76 ± 4.72	36.83 ± 3.21

Table S3. RNA-seq data statistical table

Samples	Clean reads	Clean bases	GC Content [a]	%≥Q30 [b]
CK1	20,112,488	6,020,967,256	51.01%	93.29%
CK2	21,414,419	6,407,042,530	50.43%	94.22%
CK3	22,405,876	6,704,773,296	50.34%	94.34%
CK4	19,738,605	5,908,490,034	50.90%	92.94%
Met1	19,445,016	5,821,271,970	50.70%	92.80%
Met2	20,945,678	6,266,761,508	50.27%	94.85%
Met3	21,245,811	6,356,994,960	50.19%	94.39%
Met4	22,353,601	6,688,410,332	50.05%	94.39%

[a] GC content: Clean Data GC content, the percentage of G and C bases in Clean Data;

[b] %⩾Q30: Clean Data Percentage of bases with a quality value of 30 or greater.

Table S4. Metformin significantly modulate apoptotic genes

Gene ID [a]	Gene symbol	Function	Expression level (mean FPKM [b])		Regulated
			CK	Met	
1482	*NKX2-5*	Affects the formation and development of the heart	0.84 ± 0.24	0.37 ± 0.13	down
84707	*BEX2*	Binding to DNA and facilitating transcription	0.61 ± 0.17	4.45 ± 0.71	up
79094	*CHAC1*	Regulates glutathione levels and oxidation balance	1.22 ± 0.24	15.81 ± 1.75	up
384	*ARG2*	Involved in nitric oxide and polyamine metabolism	2.81 ± 0.31	6.88 ± 0.95	up
1649	*DDIT3*	Affects lipogenesis and erythropoiesis;<br>Activated by ER stress promotes apoptosis	8.42 ± 1.80	38.49 ± 4.05	up
1907	*EDN2*	Involved in high blood pressure and ovulation processes	5.20 ± 0.74	1.73 ± 0.34	down
8061	*FOSL1*	Regulates cell proliferation, differentiation, and transformation	3.67 ± 0.58	8.61 ± 2.53	up
22822	*PHLDA1*	Involved in the anti-apoptosis of insulin-like growth factor-1	5.07 ± 0.52	11.87 ± 0.49	up
1491	*CTH*	Encodes the enzyme that converts cysteine to cysteine	6.90 ± 0.38	14.17 ± 0.45	up
51129	*ANGPTL4*	Inhibits vascular growth and tumor cell invasion	1.38 ± 0.13	3.06 ± 0.32	up
9518	*GDF15*	Involved in the stress response of cells after cell injury	25.18 ± 1.99	83.46 ± 8.20	up
50486	*G0S2*	Involved in external apoptosis signaling pathways and their forward regulation	0.81 ± 0.26	5.27 ± 1.52	up

7161	*TP73*	Involved in cellular responses to stress and development	1.15 ± 0.21	0.44 ± 0.06	down
8739	*HRK*	Involved in activating or inhibiting the apoptosis process, interacting with BCL-2 and BCL-X(L) to promote apoptosis	0.50 ± 0.14	1.13 ± 0.06	up

[a] Gene ID, Gene IDs in the NCBI database.

[b] FPKM stands for Fragments Per Kilobase of transcript per Million mapped reads.

Table S5. The signaling pathway in which key genes are located

Number	Human genes	Gene ID [a]	KEGG enrichment analysis
1	*HRK*	8739	APOPTOSIS
2	*DDIT3*	1649	PRION DISEASE、PROTEIN PROCESSING IN ENDOPLASMIC RETICULUM、APOPTOSIS、MAPK SIGNALNG PATHWAY、NON-ALCOHOLIC FATTY LIVER DISEASE、ALZHEIMER DISEASE、PARKINSON DISEASE、TRANSCRPTIONAL MISREGULATION IN CANCER、AMYOTROPHIC LATERAL SCLEROSIS
3	*PPP2R5C*	5527	PI3K-AKT SIGNALING PATHWAY、SPHINGOL IPID SIGNAL ING PATHWAY、mRNA SURVEILLANCE PATHWAY、DOPAMINERGIC SYNAPSE、OOCYTE MEIOSIS、AMPK SIGNALING PATHWAY、HUMAN PAPILLOMAVIRUS INFECTION、ADRENERGIC SIGNALING IN CARDIOMYOCYTES
4	*PPP2R5A*	10325	PI3K-AKT SIGNALING PATHWAY、SPHINGOL IPID SIGNAL ING PATHWAY、mRNA SURVEILLANCE PATHWAY、DOPAMINERGIC SYNAPSE、OOCYTE MEIOSIS、AMPK SIGNALING PATHWAY、HUMAN PAPILLOMAVIRUS INFECTION、ADRENERGIC SIGNALING IN CARDIOMYOCYTES
5	*RRAGA*	10670	mTOR SIGNALING PATHWAY、AUTOPHAGY.ANIMAL

[a] Gene ID, Gene IDs in the NCBI database.

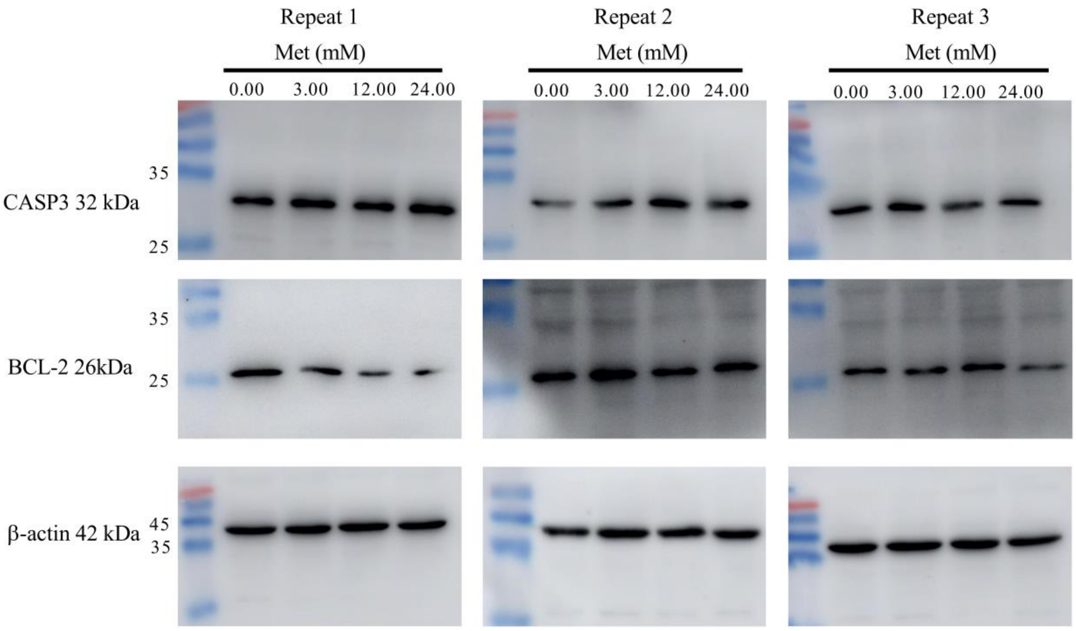

Figure S1. The triplicates for each western blot in Figure 3A.
